# Another Opinion on Celluloid

**Published:** 1883-10

**Authors:** T. C. Howe

**Affiliations:** Nebraska.


ARTICLE VIII.

### ANOTHER OPINION ON CELLULOID.

BY DR. T. C. HOWE, NEBRASKA.

My experience in the use of celluloid does not exactly correspond with the statement of Wr. R. Hall in the May number of your journal, which is just received. I will admit that it requires far more mechanical skill to manipulate than rubber, but the beauty of the work when artistically finished, more than compensates for the extra amount of care and labor required. As to durability I think it equals, if it does not excel, that of rubber. I have now used it for about 3½ years. The first two years I experimented in various ways to obtain best results. In my method of using now, good plaster casts are not forced out of place, only in exceptional cases of carelessness; nor do I meet with the trouble in repairing old plates that is such a terror to so many practitioners. I cannot induce any of my patients to accept of rubber work, even at reduced prices, after seeing my celluloid samples. I will give my method of working celluloid, in the hope that it may be of use to some that wish to use it, but are inclined to abandon it, discouraged by frequent failures. I use plain teeth universally; do not think gum teeth are adapted to celluloid work. After my cast is properly dressed up, I sift a little French chalk rub and it in thoroughly, blowing off that remaining loose. This prevents particles of base-plate adhering, also leaves the finished plate with smooth, clean surface. I wax up the teeth a little heavier than I require the plate, to be able to dress off all inequalities, thereby guaranteeing a finely polished surface that can readily be cleaned by the wearer. In investing my model in the lower half of flask, I fill up with plaster just flush with the lower edge of base-plate at labial portions, making good condyles; filling remainder of flask in the usual manner. After separating flask and cleaning teeth of wax see that the teeth are firm in the plaster, as plain teeth are much more liable to drop out than gum teeth. If by inverting upper half of flask and shaking any of the teeth drop out, place a drop or two of sandarach in the depression and firmly press tooth to place, allowing varnish to set and all will be well. Before I took this precaution, when I removed the plate from the flask I have sometimes found a single tooth imbedded in palatal portion of plate. When using metal air chamber, after filling, I rub French chalk over the surface to be exposed and fasten in place. Frequeutly I cut out a chamber in the impression and have one ready formed on the cast. If the base-plate is extra thick in any part of alveolar portions, I selected celluloid blanks that fit the cast and of half sizes, as 3½, and 4½, etc. If the palatine arch be high, have blank enough broader than cast to allow palatal part to be brought down to the model before actual pressure begins. I use dry heat, in the Ransom & Randolph old style Vulcanizer, which has a screw in the cover of the boiler for closing the flask. Into this cover I had a strong styrrup placed (made of Swedes iron) which holds my flask suspended in the boiler and not coming in contact with any part of it, insuring me a perfectly even heat. I commence to slowly close my flask at 280°, but allow it to reach 340° before perfectly closing. By means of the styrrup in the cover it can be taken off and examined to see if the flask is perfectly closed; if not, it can be returned and closing perfected.

I never use water to cool Vulcanizer in celluloid work, giving it at least twelve hours to cool.

In repairing, prepare same as for rubber; fit a piece of celluloid as nearly as possible to dove-tailed part, being sure to have quite a little surplus. After flasking, cleaning thoroughly, etc., thoroughly wet the part exposed with strong spirits of camphor, allowing a little time for it to be absorbed; now place your new material in position, saturate well with camphor again, and proceed as above.

In working by above method the dull brown that W. R. H. says obtains in plates in a year or two has not taken place as far as my observation has extended.—*Dental Practitioner*.

